# Why meta-regulation matters for public health: the case of the EU better regulation agenda

**DOI:** 10.1186/s12992-023-00971-4

**Published:** 2023-09-14

**Authors:** Kathrin Lauber, Eleanor Brooks

**Affiliations:** Global Health Policy Unit, School of Social and Political Science, Chrystal Macmillan Building, 15a George Square, University of Edinburgh, Edinburgh, EH8 9LD UK

**Keywords:** Meta-regulation, Impact assessment, Public policy, European Union, Commercial determinants, Stakeholder consultation, Evaluation, Regulatory oversight, Good regulatory practice

## Abstract

Meta-regulation – the rules that govern how individual policies are developed and reviewed – has not received much attention in the study of health policy. We argue that these rules, far from value-free and objective, have significant potential to shape policy outputs and, as such, health outcomes. Channelling and operationalising wider paradigms like neoliberalism, they determine, for instance, what is considered ‘good’ policy, how decisions are made, based on which evidence, and whose voices matter. Exploring an archetypal example of meta-regulation, the European Union’s Better Regulation agenda, we illustrate why meta-regulatory tools such as impact assessment, stakeholder consultation, and evaluation – and the norms that underlie their application – matter for health. In so doing, we concentrate especially on the ways in which Better Regulation may affect interest groups’ ability to exert influence and, conversely, how actors have sought to shape Better Regulation. We argue that attention to meta-regulation contributes to counter-balancing the focus on agency within debates at the intersection of globalisation and health, and notably those on regulatory practices and coordination. Whilst research has noted, for instance, the origins of frameworks like Better Regulation and the increasing inclusion of 'good regulatory practice' provisions within trade and investment agreements, less attention is directed to the role that these frameworks play once institutionalised. Yet, as we illustrate, there is considerable scope for meta-regulation to enhance our understanding of the forces shaping health policy via, for instance, conceptualisations of the (social, economic, political, commercial) determinants of health. As such, we call for increased attention to the role of meta-regulation in research and practice aimed at improving human and planetary health.

## Introduction

Scholars seeking to understand what shapes population health have long recognised the need to look beyond proximal factors. A significant body of work harnesses insights from relevant fields of political science, investigating the role of actors, ideas, and politics in shaping public health policy outcomes [1, 2], and an emergent literature explores how these dynamics are shaped by distal, structural forces like neoliberalism and globalisation (e.g., [3, 4]). Drawing attention to the ways in which commercial actors prevent or undermine the development and implementation of effective, equitable policies has been a particularly valuable contribution of the fast-growing commercial determinants literature [5–8]. A focus on agency, however, has often neglected the role of the wider structures within which actors operate [4]. In this Debate, we seek to highlight a specific type of structural factor which, we argue, has significant potential to enhance our understanding of the forces shaping health policy: meta-regulation [9].

Conceptually situated between the level of concrete, sector-specific regulations, and abstract, overarching paradigms, meta-regulation refers to ‘the rules that govern the rules’ [9]. Meta-regulations are the institutional structures which scaffold interactions and decision-making across all policy sectors within a political system. Often channelling and operationalising wider paradigms like neoliberalism, these rules play a crucial role in shaping policy processes. They determine, for instance, what is considered ‘good’ policy, how decisions are made, based on which evidence, and whose voices matter. These factors, in turn, have a profound impact on policy outputs. Crucially, meta-regulation shapes, and is shaped by, agency. It is a vehicle for political agendas and interest representation and, not least because of its capacity to redistribute power in the policy process, subject to lobbying like any other public policy.

Meta-regulation has rarely been problematised in the public health context. Yet, it has clear relevance to debates at the intersection of globalisation and health, notably those on regulatory practices and coordination. Meta-regulation aimed at ‘bettering’ the quality of policymaking processes and outputs is now commonplace, driven in part by the Organisation for Economic Cooperation and Development’s promotion of ‘good regulatory practice’ [10], and research documents the increasing inclusion of such provisions in new generation trade and investment agreements [11, 12]. Less attention is directed to the role that these frameworks play once institutionalised. They are rarely considered within theorisations of the (social, economic, political, commercial) determinants of health, despite their relevance to the practice of policymaking and, ultimately, policy outputs. Elsewhere, we have integrated meta-regulation into conceptualisations of ‘regulatory chill’ found in the trade and health literature, elaborating an alternative pathway by which health regulation might be weakened or precluded [13]. There remains considerable scope for attention to meta-regulation to enhance our understanding of, for instance, how corporate agency is exercised in, and impacts on, health policymaking [5, 7, 8] and how neoliberal paradigms are enshrined in the institutional mechanisms that shape policy spaces [4, 14].

In this Debate we introduce, and argue for greater attention to, meta-regulation. To demonstrate its importance to public health – which, we assert, is rooted in its interaction with agency – we present a prototypical example of meta-regulation: the European Union’s (EU) Better Regulation agenda. In so doing, we build on a body of research evidencing the role of corporations and governments in promoting Better Regulation [15, 16]. We reach beyond this to illustrate how, once institutionalised, Better Regulation enables and constrains actors’ ability to influence the health policy process, and how actors, in turn, seek to shape this infrastructure. We draw on primary analyses of EU transparency data, policy documents, and publications by key interest groups, as well as a review of the academic and grey literature, the latter including reports from non-governmental organisations and think tanks.

### Meta-regulation, agency, and public health

Bronwen Morgan defines meta-regulation as a “set of institutions and processes that embed regulatory review mechanisms into the every-day routines of governmental policymaking” [9] in the form of a sector-neutral governance mechanism. As she notes, while it is “mostly an affair of technical bureaucratic minutiae”,

The stakes underlying meta-regulation are neither technical nor dry. In essence, meta-regulation manages the tensions between the ‘social’ and ‘economic’ goals of regulatory politics, tensions that enflame passionate and highly wrought political conflict over the ethical limits of global capitalism. [9]

Meta-regulation sets the course of decision-making and many of the parameters within which those seeking to shape policy operate, including, for instance, which department leads on a particular file, at what point the public and affected stakeholders should be consulted, and how and when impacts should be assessed. It is for this reason that various national governments and multinational companies, including British American Tobacco (BAT), worked to promote mandatory impact assessment (IA) in EU policymaking [16], and have sought to extend or export 'good regulatory practice' frameworks via trade agreements like the Transatlantic Trade and Investment Partnership [17]. Yet, we know little about how meta-regulatory structures and norms – for example, the role and purpose assigned to IA, the specific assessment criteria and methodologies used, the design of oversight mechanisms – shape actors’ engagement with the policy process. These factors represent the kind of distal, structural forces that determinants of health models seek to capture and that are currently under-explored in the public health literature.

### The EU Better Regulation agenda

Better Regulation is a textbook example of meta-regulation: applicable to all policy sectors, it sets out the precise procedures that EU officials must follow when creating or revising policies, the principles and objectives that should inform policy development, and the role of particular actors and tools at different stages of the policy process. Having emerged in the United States, the idea of ‘better’ or ‘smart’ regulation gained popularity in (former) EU member states – mostly notably the UK, Germany, and the Netherlands – through the 1990s, and the adoption of an EU Better Regulation agenda was subsequently promoted by these governments, along with an array of corporate actors [16]. For the former, the agenda was a way to impose some control over the legislative initiative of the EU [18, 19]; for the latter, as illustrated by internal documents from BAT, support was rooted in a view that it “could help the company defeat efforts to introduce policies restricting smoking” [16]. The EU Better Regulation framework has evolved incrementally but the consolidated, comprehensive iteration that structures current policymaking was adopted in 2015 and amended in 2019 and 2021 [20–22].

Better Regulation seeks to improve the quality of EU legislation by strengthening its evidence base, increasing participation in policymaking, and reducing the burden of legislation for businesses and citizens [20]. In practice, it is a physical document – a ‘manual’ for policy-makers – describing the policy process, relevant tools, procedures, and responsibilities [23, 24]. Most existing work focuses on the stated aims and constituent tools of Better Regulation, assessing whether they perform *as intended* [25–27]. Less attention is paid to its underlying assumptions or wider impacts. Yet, Better Regulation is also an idea, given substance as a set of high-level guiding principles about the ends that all policies should serve. Specifically, it is a *chameleonic* idea [28]; some perceive it is a commitment to deregulate, whilst others see an effort to address the perceived lack of democratic legitimacy in EU policymaking by ensuring high-quality policy processes [29, 30]. Consequently, and like most meta-regulatory programmes, Better Regulation is difficult to pin down for the purposes of analysis. Below, we deconstruct the agenda to identify five core policy tools, rooted in the overarching norms of participation, burden reduction, and evidence-based policymaking, and supported by the application of several principles [27, 31].

### Normative basis

The reduction of regulatory burden or ‘red tape’ for citizens and businesses, including via simplification of existing interventions and mandated consideration of alternatives to regulation, has been a core aim of Better Regulation since its inception. Despite its origins and the explicitly deregulatory thrust of early iterations, the Commission asserts that the agenda “is not about regulating or deregulating” [24] but concerns regulatory quality. A second norm of participation reflects the assumption that policy is ‘better’ and more legitimate if the views of affected stakeholders and citizens are sought and considered throughout the policy process. Lastly, a norm of evidence-based policymaking is operationalised through the systematic assessment of available evidence and, preferably, quantification of policy impacts – including costs and benefits – before and after the adoption of legislation.

### Policy tools and principles

At its core, Better Regulation consists of a set of five policy tools, concrete procedural instruments which operationalise the norms set out above. These are: impact assessment, evaluation, consultation, quality control, and regulatory stock management. Notably, the Better Regulation agenda is not enshrined in EU law. While the application or outcomes of IA, evaluation, or consultation can be invoked to defend or question the legitimacy of legislation, no legal basis exists on which legislation could be dismissed for not ‘complying with’ Better Regulation.

#### Impact assessment

Key to operationalising norms of evidence-based policymaking and burden reduction, a mandate to assess some impacts of EU legislation has been in place since 1986 [32]. The 2002 Better Regulation package combined economic, social, and environmental IA into an ‘integrated’ process, and the 2015 reform saw an expansion of IA requirements to all legislative and non-legislative acts, as well as significant delegated and implementing measures [22, 23]. Some IAs are supported by risk assessments.

#### Evaluation

Evaluations of an individual intervention, or cross-cutting Fitness Checks of multiple related interventions, are used to assess the performance of initiatives against the criteria of efficiency, effectiveness, coherence, relevance, and EU added value. They also directly address ambitions to develop 'evidence-based' and minimally burdensome policy by providing baseline scenarios of costs and impacts [24].

#### Stakeholder consultation

Operationalising a norm of openness and participation, the Commission uses a broad range of methods to consult stakeholders throughout policy preparation and review. Consultation can take the form of public questionnaires, conferences, targeted meetings, or invitations to comment on documents. The aim is to provide all interested and affected parties with the opportunity to contribute their views on IAs, major evaluations and Fitness Checks, draft delegated and implementing acts, adopted policy proposals, and ongoing opportunities for simplification. Later reforms introduced an additional consultation stage, based on ‘calls for evidence’ (previously Roadmaps or Inception IAs), which prompts stakeholder input at an early point in the policy process [22, 33].

#### Quality control

Regulatory oversight is intended to uphold the ‘quality standards’ set by the Better Regulation agenda. The Regulatory Scrutiny Board (RSB, previously Impact Assessment Board) is led by a Director-General and staffed with three external experts and three high-level Commission officials on a three-year mandate [34]. It assesses the quality (not the substantive content) of all IAs and major evaluations, and advises on Better Regulation more broadly. RSB opinions are not formally binding, but IAs are not deemed adequate in the absence of a positive opinion, and a second negative opinion means that initiatives can only progress if the relevant Vice-President permits [23].

#### Regulatory stock management

Finally, the Better Regulation agenda implements several instruments designed to control the stock and flow of EU regulation. The REFIT (regulatory fitness and performance) programme structures systematic review of existing laws, to ensure that they are ‘fit for purpose’ and that simplification and burden reduction potential is considered throughout the policy cycle [24, 35]. REFIT is complemented by the Commission’s new one-in, one-out (OIOO) programme, which seeks to “[offset] newly introduced burdens […] by removing equivalent burdens in the same policy area” [36, 37].

### Principles

Throughout the application of the above policy tools, a set of cross-cutting principles guide decision-making. Proportionality and subsidiarity establish that EU action should not go beyond what is necessary to achieve the desired outcome and should not proceed unless it is more effective than action at the national level, respectively. They are codified as Treaty law but were developed within the debates that underpinned early Better Regulation initiatives and have an inherent concern with “restraint in both the form and content of regulation” [38]. Several non-Treaty principles have been integrated into the Better Regulation toolbox through specific criteria and guidance, although the consistency of their application remains unclear [39]. Such principles include ‘think small first’ (focusing on small and medium enterprises), mainstreaming EU action in pursuit of the Sustainable Development Goals, ‘digital by default’ (advancing the EU’s ‘digital transition’ policy), operationalising the European Green Deal pledge to ‘do no significant harm’ to environmental objectives, and integrating ‘strategic foresight’ into decision-making [23, 33]. Of particular note here is the innovation principle which commits the EU to systematically consider impacts on firms’ “capacity and incentives to innovate” [23] when developing policy [40, 41]. Favoured by corporate actors, this principle is often presented as ‘complementary’ to the precautionary principle [42], which posits that risk management action should be taken despite scientific uncertainty if there are reasonable grounds to anticipate serious or irrevocable impacts on human or planetary health, and is widely recognised as an important driver of progressive EU environmental protections. Although the innovation principle is not part of Treaty law like the precautionary principle, its growing uptake calls for heightened scrutiny of its implications for health and environmental regulation [43, 44].

### Agency shapes structure shapes agency: the role of Better Regulation in health policymaking

The norms, tools, and principles constituting Better Regulation *shape* and *are shaped by* agency, interacting with the practice of policy-makers, interest groups, politicians, and other actors. Focusing on corporate agency, this section illustrates the pathways through which stakeholders act on Better Regulation, and how the agenda, in turn, shapes their advocacy efforts.

### Lobbying ‘one level up’: how have actors sought to shape Better Regulation?

The shaping of meta-regulation by stakeholders is an example of lobbying ‘one level up’*,* beyond the content of sector-specific proposals. In the campaign to introduce Better Regulation at the EU level, BAT capitalised on the chameleonic nature of the idea and adapted its framing of Better Regulation according to its audiences. When speaking to other large corporations, BAT and its wider coalition described Better Regulation as a fundamental regulatory reform; when writing in papers aimed at policy-makers, it was framed more narrowly, as a technical tool to improve the quality and legitimacy of decision-making [16]. Two decades later, a think-tank associated with BAT and involved in these papers, the European Risk Forum (now the European Regulation and Innovation Forum, ERIF), lists among its self-declared achievements the important role it played in the adoption of the innovation principle, the implementation of detailed evaluation guidelines, and the establishment of the RSB [45]. The Forum, whose members include a range of companies, public relations firms, and business associations, remains active in its efforts to shape EU regulatory practice. In addition to its well-established role in promoting the innovation principle against a backdrop of concern about the “disproportionate of unjustified use of precaution” [46], recommendations made in its published materials include, for instance, extending requirements for consultation and IA, and assessing ‘ideological’ of ‘belief-based’ bias alongside financial conflicts of interest in regulatory expert committees [47]. Similarly, demands raised by BusinessEurope, a multi-industry lobby group, include deeper integration of the innovation principle, and an overall greater emphasis on minimising burdens on business, including via the new OIOO approach (e.g., [48–50]).

Several major civil society organisations have (more recently) included Better Regulation in their advocacy and launched counter-campaigns which highlight the potential threat that the current iteration of Better Regulation poses to health and environmental protections [51–54]. Responding with alarm to the 2015 reforms, a civil society coalition launched the Better Regulation Watchdog, aimed at resisting the weakening and neglect of essential regulations [55]. However, the initiative has since fallen inactive and, overall, it appears that corporate actors continue to be more successful in shaping the Better Regulation agenda, directly and indirectly [16]. Available meeting disclosures from high-level Commission officials and the RSB suggest that the sustained outreach by companies and business groups manifests in more access, and indicate that between 2015 and 2022, no external organisation had as many meetings on the subject of Better Regulation as BusinessEurope, closely followed by the American Chamber of Commerce to the EU [56].

Although corporate actors’ support for and role in establishing Better Regulation does not necessarily mean that the agenda is counterproductive for health or the environment, awareness of the ways in which interest groups have attempted and continue to shape the agenda – and why – is crucial for recognising its potential implications.

### How does better regulation shape actors’ capacity to influence policy?

Using illustrative examples, we outline two interconnected pathways between Better Regulation and the institutional power of corporate actors, in particular: *first*, Better Regulation can be used instrumentally through engagement with the tools described above; *second,* the norms and principles of Better Regulation can be employed discursively.

### Instrumental use of better regulation tools

There is a risk that IA requirements for specific evidence provide ripe grounds for informal challenge on procedural grounds. A well-known example is the case of REACH (Registration, Evaluation, Authorisation and Restriction of Chemicals), a major regulation which applies to all chemical substances. When the Commission announced that it was developing REACH in 2003, industry groups commissioned several consultancies (including the company later tasked with conducting the Commission IA) to produce alternative assessments, emphasising regulatory costs for business, and successfully lobbied for the inclusion of three industry-sponsored assessments within the Commission’s IA framework [15, 57]. More recently, the Commission’s proposal for a revised packaging and waste regulation (Regulation (EU) 2019/1020) was followed by a campaign led by McDonald’s, opposing the focus on packaging reuse [58]. Titled ‘No Silver Bullet’, the campaign was based on a report which was positioned as an impact assessment study. Authored by consulting firm Kearney for McDonald's, it concludes, inter alia, that reuse models can lead to high costs and increased plastic waste, arguing instead that “[s]takeholders across the value chain – including private, public, and civil sectors – need to work together to develop a set of mixed solutions” [59].

Further, the requirement to conduct increasingly complex IAs risks delaying much needed action, which can be amplified by challenges and procedural issues, such as RSB rejection [14, 15, 60]. The role of the RSB in ‘enforcing’ Better Regulation and quality-controlling policy development has also been criticised on the basis that the Board’s membership has included little health or environmental expertise, its independence is stated but not safeguarded, and its interactions with stakeholders are not sufficiently transparent [53, 61, 62]. A recent report, for instance, described the RSB, and Better Regulation more generally, as “a self-inflicted obstacle to re-protecting Europe” [61].

Far from a value-neutral tool to gather feedback, consultation frequency, form, design, and analysis intrinsically shape actors’ engagement with decision-makers. While the expansion of consultation under Better Regulation theoretically enables a wide range of societal actors to feed into decision-making, in practice, it also risks privileging well-resourced business interests that are better-able to translate increased opportunities into access [15, 52, 60]. In the recent consultation on the revision of the General Pharmaceutical Legislation (GPL), for instance, nearly half of the feedback received on the early-stage inception IA and roadmap document (48.5%) originated from companies or business associations, and the pharmaceutical industry made up 28.4% of respondents to the open public consultation. In a targeted survey used to gather specific feedback, the Commission acknowledged that “[r]epresentation amongst the different groups was not as anticipated with industry particularly over-represented (55.1%) and [civil society organisations] underrepresented (5,8%)” [63]. Moreover, available information suggests that pharmaceutical industry groups were able to meet significantly more frequently with officials. Commission Vice-President Margaritis Schinas and his cabinet, for instance, engaged in at least 26 meetings between January 2020 and October 2023, 22 of which were solely with pharmaceutical companies or industry associations [64, 65]. Whilst a causal link between engagement in consultation and influence over policy is far from certain, the imbalance in access afforded through Better Regulation tools is important to account for when analysing the policy process.

At the time of writing, implementation of OIOO is underway [66]. Proponents of regulatory offsetting, predominantly consisting of businesses and some member states, have expressed concerns that the initiative is “too little” (too narrow in scope and overly flexible) and comes “too late” (following large packages such as ‘Fit for 55’) [67]. Opponents, on the other hand, take issue with the potential risk of neglecting societal benefits when calculating poorly defined burdens and providing a pathway to challenge legislation on the basis that it would increase costs [53, 67]. Moreover, OIOO necessitates the quantification of costs and benefits [66], known to exacerbate decision-makers’ dependence on input from industry (which is better placed to offer quantitative calculations of costs) and the intrinsic methodological difficulties in predicting, measuring, and quantifying longer-term benefits of regulation [60, 68]. The Commission’s summary of the consultation process for the GPL revision, for instance, states that “most stakeholders interviewed could not provide specific quantitative estimates”, but goes on to note that industry stakeholders provided information on costs and “a few industry respondents to the survey provided one-off adjustment costs, related to […] ongoing regulatory costs” [63].

### Discursive use of better regulation norms and principles

The EU’s own narratives around Better Regulation can be used strategically by those seeking to shape policy. For instance, the prevailing emphasis on burden reduction – coupled with the absence of concrete detail regarding what constitutes (un)acceptable costs for protecting health and the environment – invites framing of *bona fide* regulations as red tape to be cut [51, 52, 61, 67].

Perhaps owing to their greater interest in the agenda, corporate actors more commonly invoke Better Regulation in attempts to block, delay, or weaken regulatory action, whereas civil society groups do not routinely draw on it. Research documents this trend in tobacco, human rights and environmental legislation (e.g., [61, 69, 70]). More recently, in consultations for EU initiatives to improve diets, for example, discursive use occurred almost solely in submissions by business groups and companies, which invoked Better Regulation to encourage consideration of soft law alternatives to regulation, comprehensive assessment of business impacts of regulation, and avoiding adding to the regulatory stock (e.g., [71, 72]). In the GPL revision, for instance, pharmaceutical companies used the public consultation to note that,

“[…] minimisation of bureaucratic hurdles is crucial and imposing additional regulatory or financial burden on industry, especially small and mid-sized companies, should be avoided to ensure attractiveness of Europe as an environment for manufacturing, [research and development] and innovation.” [73].

Similarly, in its response to the consultation on an EU sustainable food systems framework, the World Federation of Advertisers refers to Better Regulation to support calls for self- and co-regulatory governance models:

“Advertising self-regulation and self-regulatory codes should therefore continue to be recognised within the future legal framework and must be accounted for in any legislative initiative, in accordance with the […] EU Commission’s Better Regulation Agenda” [74].

Importantly, discursive use of Better Regulation norms by those who oppose stricter rules may stymie the development of *bona fide* regulation at earlier stages [14, 60]. In line with theories of regulatory chill [75], the anticipation of challenges on political or technical grounds may prompt policy-makers to adjust the options considered and the ambition of resulting proposals [13].

### Conclusions: accounting for the rules that govern the rules that determine health

Reflecting the important role of regulation in protecting populations from harmful products and practices, understanding the ways in which political actors influence regulatory policies is increasingly recognised as key to advancing public health [76]. In reviewing the role and relevance of the EU’s Better Regulation agenda we hope to have demonstrated the need to look beyond specific policy areas, to meta-regulation, and to actors’ ability to shape and use such institutions in their favour (what Barnett and Duval [77] term *institutional power*). Attention to the wider policymaking infrastructure is critical to our understanding of what may have to change *upstream* to support a policy environment that fosters equitable health outcomes. The case of EU Better Regulation illustrates that further empirical and conceptual work is needed to characterise the role of meta-regulation in shaping pathways for influence and policy outputs.

Analysis of the EU Better Regulation case reveals the insight that the tools of political science and adjacent fields can offer when applied through a critical public health lens. The role of interest groups in promoting and shaping the Better Regulation agenda, for instance, reminds us that politics behind the choice of governance tools merit careful scrutiny. Concepts like instrument constituencies [78] and informal governance [79] have much to offer our understanding of why particular tools prevail and perform as they do. Similarly, the intended and unintended impacts of meta-regulation across all aspects of policymaking – actions, norms, knowledge – could be better understood through a focus on practices [80, 81]. More fundamentally, the discursive use of Better Regulation suggests a need to complement the study of institutions with analysis of the ideas and discourses that shape meta-regulatory environments [82, 83]. Although positioned as striving towards a neutral concept of quality, the ‘better’ in Better Regulation reflects one of many, sometimes conflicting, views of what ‘good’ regulation and governance look like [57, 84]. This has been core to actors’ efforts to shape Better Regulation for different purposes as well as its strategic invocation in policy debates, a dynamic which can only be understood by exploring how ideas are created, maintained, challenged, and utilised in policy processes.

In sum, the *rules that govern the rules* are a crucial but often overlooked element of the political systems and mechanisms that shape public health. In particular, a better understanding of lobbying and wider efforts to shape policy through the lens of meta-regulation, supported by closer engagement with the political science toolbox, has the potential to meaningfully contribute to the improvement of governance for public health.

## Data Availability

All material used in this article is publicly available and referenced.

## Abbreviations

BAT: British American Tobacco
ERIF: European Regulation and Innovation Forum
EU: European Union
GPL: General Pharmaceutical Legislation
IA: Impact assessment
OIOO: One in, one out
RSB: Regulatory Scrutiny Board
REACH: Registration, Evaluation, Authorisation and Restriction of Chemicals
